# Common Process Demands of Two Complex Dynamic Control Tasks: Transfer Is Mediated by Comprehensive Strategies

**DOI:** 10.3389/fpsyg.2017.02145

**Published:** 2017-12-19

**Authors:** Wolfgang Schoppek, Andreas Fischer

**Affiliations:** ^1^University of Bayreuth, Bayreuth, Germany; ^2^Forschungsinstitut Betriebliche Bildung, Nürnberg, Germany

**Keywords:** complex problem solving, complex dynamic control, dynamic decision making, strategies, knowledge acquisition

## Abstract

Although individual differences in complex problem solving (CPS) are well–established, relatively little is known about the process demands that are common to different dynamic control (CDC) tasks. A prominent example is the VOTAT strategy that describes the separate variation of input variables (“Vary One Thing At a Time”) for analyzing the causal structure of a system. To investigate such comprehensive knowledge elements and strategies, we devised the real-time driven CDC environment Dynamis2 and compared it with the widely used CPS test MicroDYN in a transfer experiment. One hundred sixty five subjects participated in the experiment, which completely combined the role of MicroDYN and Dynamis2 as source or target problem. Figural reasoning was assessed using a variant of the Raven Test. We found the expected substantial correlations among figural reasoning and performance in both CDC tasks. Moreover, MicroDYN and Dynamis2 share 15.4% unique variance controlling for figural reasoning. We found positive transfer from MicroDYN to Dynamis2, but no transfer in the opposite direction. Contrary to our expectation, transfer was not mediated by VOTAT but by an approach that is characterized by setting all input variables to zero after an intervention and waiting a certain time. This strategy (called PULSE strategy) enables the problem solver to observe the eigendynamics of the system. We conclude that for the study of complex problem solving it is important to employ a range of different CDC tasks in order to identify components of CPS. We propose that besides VOTAT and PULSE other comprehensive knowledge elements and strategies, which contribute to successful CPS, should be investigated. The positive transfer from MicroDYN to the more complex and dynamic Dynamis2 suggests an application of MicroDYN as training device.

## Introduction

Complex problem solving (CPS) is a phenomenon that is investigated in many domains, ranging from scientific discovery learning over industrial process control to decision making in dynamic economical environments. At the heart of the scientific investigation of the phenomenon are complex dynamic control (CDC) tasks (Osman, 2010) that are simulated in the laboratory. Simulated CDC tasks provide the opportunity to study human deciding and acting in complex situations under controlled and safe conditions.

Currently, research on CPS is dominated by attempts to construe it as one-dimensional ability construct, which means that a single measure represents a person's ability to solve complex problems. To this end, Greiff and Funke (2010) and Greiff et al. (2012) have developed the minimal complex systems test MicroDYN. This CPS environment consists of a number of linear systems with mostly three input and three output variables. The systems are presented with various cover stories (e.g., how do different training schedules affect aspects of handball performance?). The subjects have to explore each system, enter their insights into a causal diagram (knowledge acquisition phase) and subsequently steer the system to a given array of target values by entering input values (knowledge application phase). Each system is attended to for about 5 min. MicroDYN yields reliable measures of knowledge acquisition and knowledge application (Fischer et al., 2015a). As both variables are highly correlated, they are often combined to obtain a measure of CPS ability (e.g., Greiff and Fischer, 2013). MicroDYN has been validated using various criteria—predominantly school grades. The typical result of these studies is that the combined CPS measure accounts for 5% variance in school grades incremental to figural reasoning (Schoppek and Fischer, 2015).

Consistent with our view of CPS as a multifaceted phenomenon (Schoppek and Fischer, 2015), we claim to use the denomination “complex problem solving” in a broader sense. We adhere to the conception of Dörner (1997), who characterizes complex problems as being complex (many variables), interrelated (with many relations among the variables), dynamic (with autonomous state changes), intransparent (with not all information being available at the outset), and polytelic (more than one goal has to be considered; often goals are contradicting). As these characteristics are not defined precisely, and can take shape to varying degrees, CPS refers to a broad range of problems, which can differ considerably in their requirements for being solved (Fischer and Neubert, 2015). This could be considered a conceptual weakness. However, for the labeling of broad phenomena this is common practice. For example, the established label “problem solving” has an even larger domain. Therefore, assuming a one-dimensional construct “CPS” does not do justice to the heterogeneity of the domain (Fischer and Neubert, 2015).

**Figure 1:**
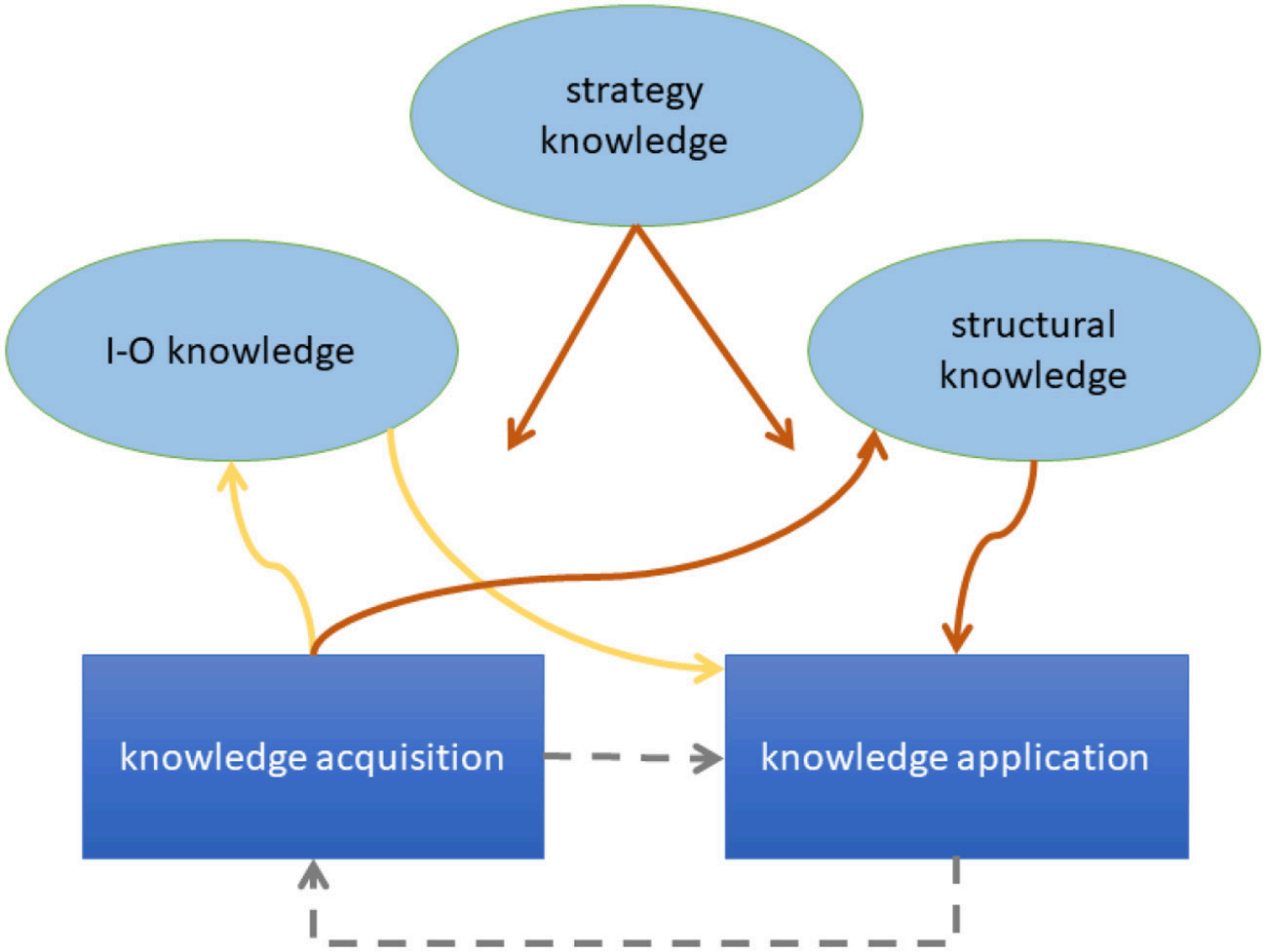
Visualization of the preliminary process model. Brown arrows denote processes that require much working memory capacity; yellow arrows denote processes that require little working memory capacity. The arrows originating from “strategy knowledge” indicate that this knowledge determines most of the displayed processes. Gray arrows indicate that the processes can iterate within the same problem.

In order to make progress toward a deeper understanding of CPS we propose a preliminary process model (see Figure 1). The model is composed of assumptions that are established in the CPS literature. We classify these assumptions as pertaining to processes and structures.

One coarse *process* assumption divides CPS in the phases (or sub-processes) of *knowledge acquisition* and *knowledge application* (Fischer et al., 2012). Knowledge acquisition refers to the requirement of detecting the causal structure of the system by means of appropriate exploration strategies^1^. Knowledge application means using the acquired knowledge to plan and implement interventions in order to reach given target states. This assumption of Fischer et al. (2012) originates in the Dynamis approach by Funke (1991, 1993) and underlies the MicroDYN paradigm (Greiff and Funke, 2009). In view of the widely spread use of this model, we call it the “standard model of CPS.” A second classification, proposed by Osman (2008, 2010), distinguishes between monitoring, which “refers to online awareness and self-evaluation of one's goal-directed actions” (Osman, 2008, p. 97), and control, which refers to “the generation and selection of goal-directed actions” (ibid., p. 97). As Osman (2008) operationalized monitoring through observation of exploration behavior of oneself or others, the kinship between monitoring and knowledge acquisition becomes obvious. However, control pertains to knowledge application *and* exploratory manipulations (which are part of the knowledge acquisition sub-process of the standard model).

With respect to *structure*, Schoppek (2002) has proposed a classification of knowledge types that are learned during and/or applied to CPS: *Structural knowledge* is knowledge about the causal relations among the variables that constitute a dynamic system. *I-O knowledge* (shorthand for “input-output knowledge”) represents instances of interventions together with the system's responses. *Strategy knowledge* represents abstract plans of how to cope with the CDC problem. An example is the awareness of the control of variables strategy (Chen and Klahr, 1999), also known as VOTAT (Vary One Thing At a Time, Tschirgi, 1980).

VOTAT was first described in the context of testing hypotheses in multivariate stories (Tschirgi, 1980). In the context of CDC tasks, it means varying a single input variable in order to observe its effects on the output variables. The extent of using this strategy predicts better structural knowledge and better control performance (Vollmeyer et al., 1996; Wüstenberg et al., 2014).

A related strategy is to apply an impulse to an input variable: The problem solver sets one or more input variables to certain values greater than zero, then sets the values back to zero again. In the following simulation steps where all input variables are zero, the course of the output variables informs the problem solver about side effects and eigendynamics of the output variables^2^. Schoppek (2002) instructed this strategy to participants in an experiment that involved a CDC task of the Dynamis type and found better structural knowledge in the trained group (see also Beckmann, 1994 and Schoppek and Fischer, 2015). Evidence about the usefulness of this strategy for controlling MicroDYN has recently been reported by Greiff et al. (2016) and Lotz et al. (2017). These authors refer to the strategy as non-interfering observation or NOTAT. We use the label PULSE, following Schoppek's (2002) characterization as setting an impulse.

Back to the process model: Processual and structural assumptions are different perspectives rather than alternative conceptions. For example, in the knowledge acquisition phase the goal is to gain structural knowledge about a system by application of appropriate strategies such as VOTAT, which are part of the strategy knowledge of the problem solver. The execution of VOTAT in turn is a process.

Our process model includes assumptions about the transfer distance of the different knowledge types (Schoppek, 2002). Structural knowledge about one specific System A cannot be transferred to another System B with a different structure (far transfer, see Paas, 1992). However, it can be transferred to the problem of reaching a different goal state in System A (near transfer). In contrast, strategy knowledge acquired in the context of System A can likely be transferred to System B. This is particularly plausible when the strategy refers to the acquisition of structural knowledge. For example, if participants learn to apply the VOTAT strategy to System A successfully, we expect them to try it also when confronted with a new System B. Such cross-situational relevance has been shown repeatedly for the VOTAT strategy (Müller et al., 2013; Wüstenberg et al., 2014). We indicate the fact that VOTAT can be applied to a wide range of problems by referring to it as a comprehensive strategy.

Further assumptions of our preliminary process model pertain to the role of working memory (WM). We assume that the various strategies that serve knowledge acquisition are differing with respect to WM requirements. A simple trial and error strategy, associated with low WM load, is not efficient for learning the causal structure of a system, but may be suitable for acquiring I-O knowledge—which is probably often memorized implicitly (Dienes and Fahey, 1998; Hundertmark et al., 2015). The VOTAT strategy on the other hand puts a heavy load on WM and is suitable for acquiring structural knowledge. To substantiate such assumptions, we adopt the terminology of cognitive load theory (Sweller, 1988; Sweller and Chandler, 1994). Solving a new complex problem yields intrinsic cognitive load. Corbalan et al. (2006) describe this to the point: “In terms of cognitive load theory the difficulty of a task yields intrinsic cognitive load, which is a direct result of the complex nature of the learning material. That is, intrinsic cognitive load is higher when the elements of the learning material are highly interconnected (…) and lower when they are less interconnected” (p. 404). Cognitive load associated with learning is called “germane load.” As the capacity of WM is limited, high intrinsic load leaves little capacity for germane load, thus leading to poor learning. Together, these assumptions predict that the difficulty and complexity of a source problem restrain the learning of generalizable knowledge about structures or strategies, leading to poor transfer. This prediction has been confirmed by Vollmeyer et al. (1996) in the context of CPS.

In summary, to learn comprehensive strategies such as VOTAT, learning opportunities should not be too complex. We suppose that transfer experiments are particularly useful for investigating the reach or comprehensiveness of knowledge elements and strategies.

To test some of the predictions of our preliminary process model, we have developed Dynamis2, a new CPS environment that accentuates the aspect of dynamics, which has been central in early work on CPS (e.g., Dörner and Schaub, 1994). Like MicroDYN, it is based on Funke's (1991, 1993) Dynamis approach, which uses linear equations for calculating state changes of the system's variables. Unlike the traditional approach, Dynamis2 simulates system dynamics in real time, which means that the state of the system is mandatorily updated every second. The user can apply inputs at any time. A typical run with Dynamis2 comprises 250 simulation steps. As much of the research with CDC tasks has been done with systems whose states are updated in less than 9 time steps—triggered by the user—we regard Dynamis2 as an important step toward investigating dynamic decision making that deserves this label (cf. Fischer et al., 2015b; Schoppek and Fischer, 2015).

The primary goal of the present study was to test assumptions about the transfer of knowledge elements, in particular strategic knowledge, from one CDC task to another. We did this with a transfer experiment where the source function and the target function of two CPS environments were completely combined^3^. This enabled us to estimate transfer effects in both directions. Secondary goals were to explore the psychometric properties of Dynamis2, and to use it as validation criterion for the more established MicroDYN (Greiff et al., 2012). MicroDYN has not been validated extensively with other standardized CPS tasks (but see Greiff et al., 2013, 2015; Neubert et al., 2015). Therefore, it appears worthwhile to test the expectation that MicroDYN predicts performance in Dynamis2 over and above intelligence. In a fashion that was common at the time when we planned the experiment, we used figural reasoning as a proxy for general intelligence. We will discuss the implication of this decision and its relation to recent findings about broader operationalizations of intelligence in the discussion section (Kretzschmar et al., 2016; Lotz et al., 2016).

We expected (1) positive transfer from MicroDYN to Dynamis2, mediated by the VOTAT strategy. As demonstrated by Wüstenberg et al. (2014), the extent of using this strategy predicts performance in MicroDYN. As VOTAT was in the focus of discussion about strategies in CDC tasks at the time when we designed the experiment, we did not explicitly expect PULSE as a mediator. However, we investigated the role of that strategy in *post-hoc* analyses. We expected (2) less to no transfer from Dynamis2 to MicroDYN, because the former is more difficult than the latter. Due to the quick time lapse of Dynamis2, the learner has to coordinate several concurrent subtasks in real time: Observing the course of the system, analyzing the effects of their actions, and planning new interventions. In terms of cognitive load theory (Sweller, 1988; Sweller and Chandler, 1994), this results in much more intrinsic cognitive load than MicroDYN, where the environment guides the course of action. Therefore, controlling Dynamis2 leaves less WM capacity open for germane load, which is necessary for conscious learning (Rey and Fischer, 2013). Based on recent evidence on the relation between CPS and intelligence (Wüstenberg et al., 2012; Greiff et al., 2013), we expected (3) that figural reasoning and MicroDYN should predict performance in Dynamis2. MicroDYN should explain unique variance in Dynamis2 (beyond figural reasoning) due to similar requirements (linear equation systems, knowledge acquisition, knowledge application).

## Methods

We first introduce the instruments and the tasks we used in the experiment, including the measures for performance and proceeding, followed by the description of the design, the participants, and the procedure. Although some of the measures were only subject to exploratory analyses, which we conducted after testing the hypotheses, we report their operationalization here.

Figural reasoning was measured with a modified version of the WMT (“Wiener Matrizentest”, Formann et al., 2011). Because the original test was constructed for adolescents, we replaced two items of the original test by four more difficult items from the original APM (Raven et al., 1994). The highest possible score was 20 points. Although matrix tests load high on general intelligence assessed with broader batteries (Johnson and Bouchard, 2005), we refer to our measure as “figural reasoning”.

*Wason task*: This task requires interactive hypothesis testing (Wason, 1960). Participants are shown a list of three numbers and are asked to find out the rule that underlies the list. For example, if the list is “2 4 6,” the rule might be “three ascending even numbers” or simply “three different numbers.” To test their hypotheses, participants enter new lists and are given feedback whether the lists conform to the rule or not. To solve problems of this kind, it is important to try to falsify one's hypotheses. Many subjects fail the task because they focus on confirming their hypotheses (Gorman and Gorman, 1984). We presented the task with three different rules. (The first was the original rule used by (Wason, 1960): “any ascending sequence”. AF devised the other two rules in the style of the first rule). As a performance measure (“Wason score”) we used the number of correctly identified rules.

### Complex dynamic control tasks

**Figure 2:**
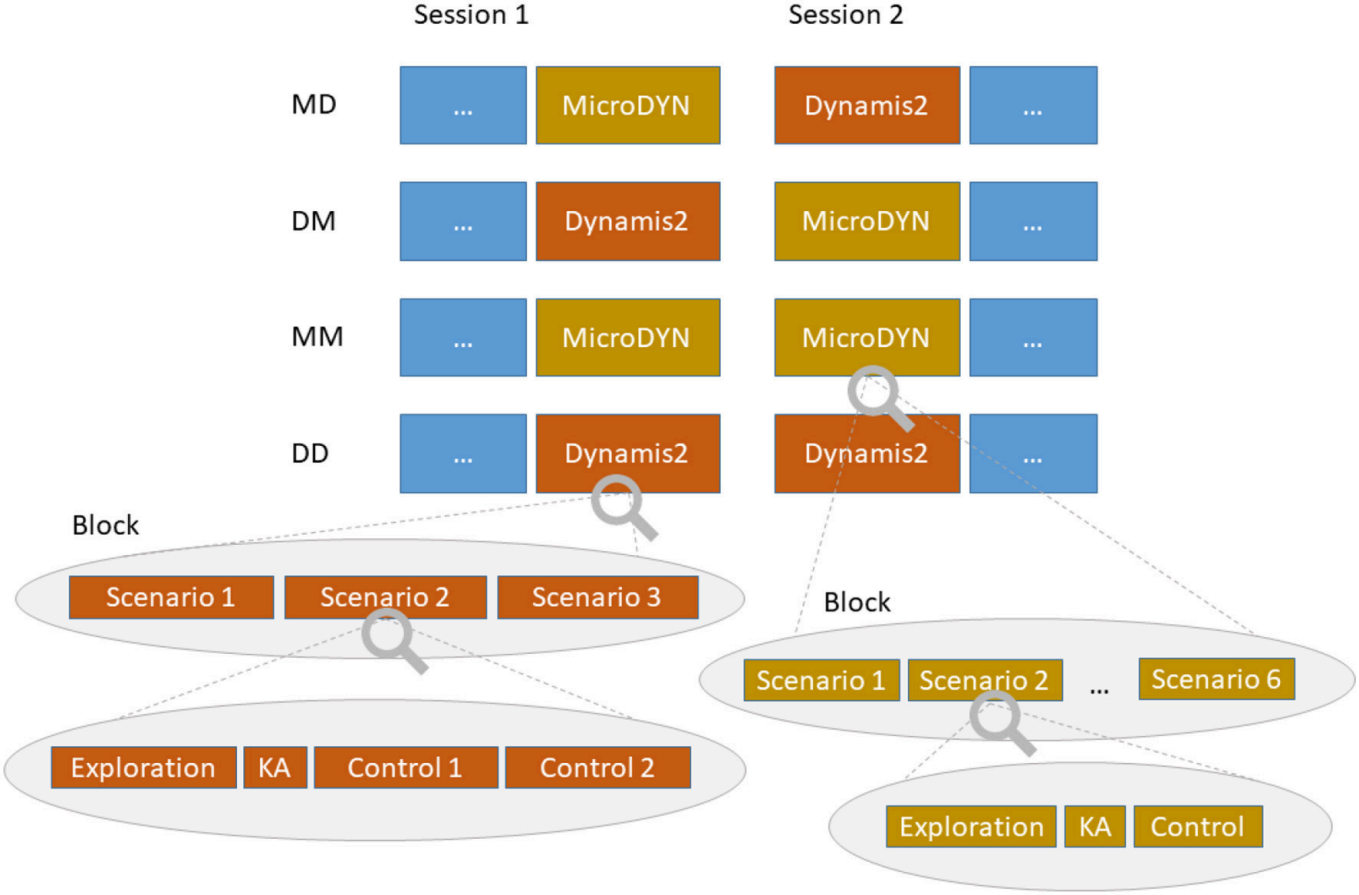
Delineation of the design and the terminology used to describe the CDC tasks. KA stands for “knowledge acquisition.”

Both CDC tasks we used in the experiment are based on linear equation systems with up to three input variables and up to three output variables (cf. Fischer et al., 2015a). The state of the system is calculated in discrete time steps as a function of the current state of the input variables and the state of the output variables from the preceding time step. We refer to these time steps as cycles. Figure 2 shows an overview of the terminology we used to describe the CDC tasks. Details about the individual systems are reported in the Appendix.

*MicroDYN*: This CDC is constructed in the style of a test, consisting of several scenarios. Each scenario is defined by a specific equation system and a corresponding cover story. The process of working on the task is the same for each scenario: First, the problem solver has to explore the system's causal structure by repeatedly varying the input variables and monitoring the effects (knowledge acquisition). To complete a cycle and see the effect of their actions the problem solver has to click a button (labeled “apply”). The problem solvers enter their insights about the systems as arrows in a causal diagram. There is a time restriction of 180 s for the exploration phase of each item. After this, the problem solvers are given goal states for each output variable that they must achieve within 90 s by manipulating the input variables up to four cycles in a row.

To assess structural knowledge, we had participants draw arrows in a causal diagram at the bottom of the screen. An arrow represented an assumed causal relation. A causal diagram was rated correct if it contained all causal relations of the system and no relation that was not simulated. Structural knowledge in the knowledge acquisition phase was scored by summing up the ternary graded degree of correctness over all causal diagrams (0: more than one error, 1: one error, 2: no errors).

Performance in the knowledge application phase was scored by summing up the ternary graded degree of target achievement across the six items (0: targets missed, 1: targets partially met, 2: targets totally met; a single target was coded as met when the deviation was no larger than ±1). As an overall performance measure, we added the knowledge acquisition score and the knowledge application score and divided the sum by two.

To determine the problem solvers' strategies, we analyzed the log files. For each cycle we observed if all input-variables were set back to zero (PULSE strategy, see below). If only one variable was set to a value different from zero at least once (VOTAT strategy), it was determined for which variable this was the case. Over all cycles of the exploration phase, we scored the proportion of input variables for which the VOTAT strategy was applied, and whether or not the PULSE strategy was applied at least once (0–1). These values were averaged across the scenarios to represent the extent of using each strategy. For example, when there are three input variables in a system and the participant used VOTAT for two of them at least once, the VOTAT measure is 0.66.

**Figure 3:**
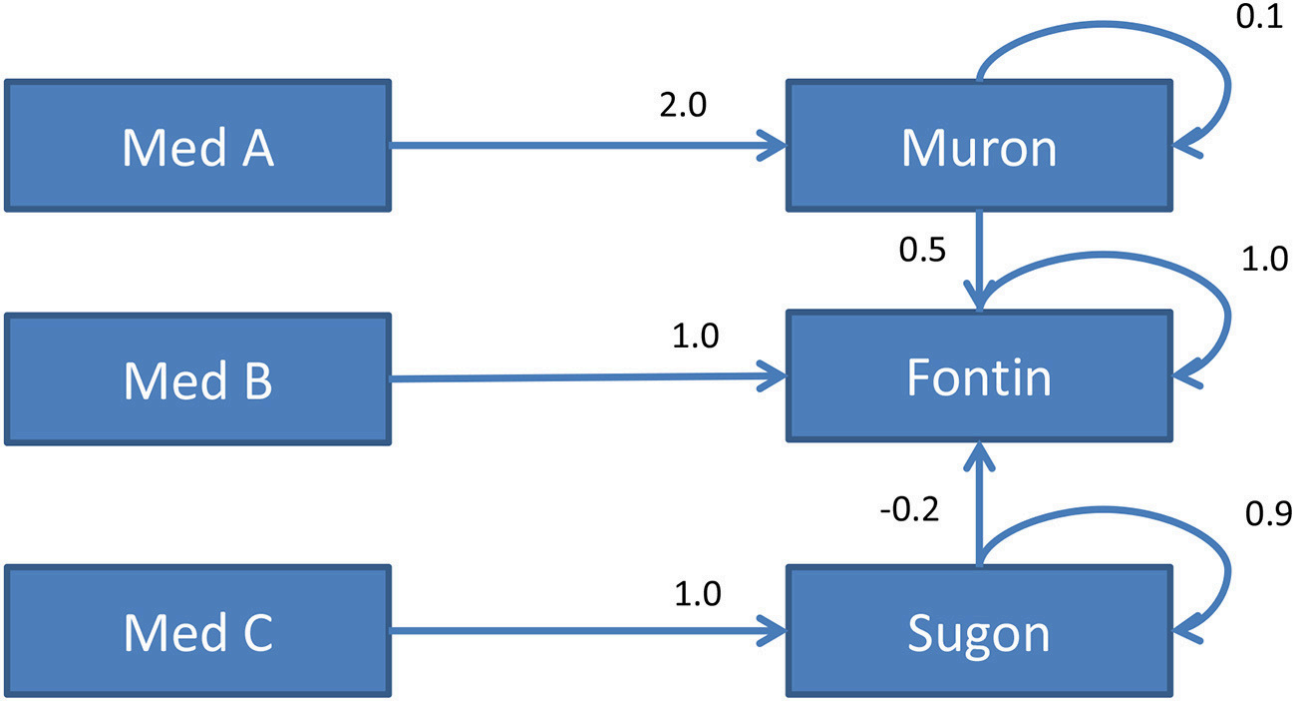
Diagram of the causal structure of one of the Dynamis2 systems used in the experiment. The numbers denote coefficients in the linear equations that determine the state of each output variable. For example, the state of Muron at time *t* is given by Muron_*t*_ = 0.1*Muron_*t*−1_ + 2.0*MedA_*t*_.

*Dynamis2* was developed in order to emphasize the dynamic aspect of complex problem solving (Schoppek and Fischer, 2015). Like in the original Dynamis approach (Funke, 1991, 1993), the systems are simulated using sets of linear equations. The crucial difference is that Dynamis2 is real-time driven, which means that the simulation is updated every second, regardless if the subject manipulates the input variables or not. This makes the dynamics of the simulated systems more tangible than in extant CPS environments such as the business microworld Tailorshop, MicroDYN, Genetics Lab, Cherry Tree (Beckmann and Goode, 2014), etc. In addition, genuine time pressure results for the subjects. Figure 3 shows the causal diagram of one of the systems used in the experiment. Subjects can manipulate the three medicines Med A, Med B, and Med C (input variables) in order to control the blood values of three fictitious substances Muron, Fontin, and Sugon (output variables). Interventions can be entered for one or more input variables and applied at any time by clicking the “apply” button. Each scenario of Dynamis2 consists of a run (250 cycles) of free exploration, followed by two runs where subjects are asked to reach and maintain a given goal state (e.g., Muron = 100, Fontin = 1,000). Performance in the goal runs is measured by goal deviation according to Equation 1, where *n* is the number of cycles (here 250), *k* is the number of goal variables, *x*_*ij*_ is the value of variable *j* in cycle *i, g*_*j*_ is the goal value of variable *j*, and *s* is the cycle when the learners entered their first input.

(1) $$dev=\ln\left(\sum_{i=s}^{n}\frac{\sum_{j=1}^{k}\left|x_{ij}-g_{j}\right|}{\sum_{j=1}^{k}g_{j}}\right)$$

Because this measure is hard to interpret, we centered it on the grand mean and reversed the scale. The resulting score thus has the same orientation as the other performance measures: Higher values represent better performance.

After completion of the exploration phase, we had participants draw arrows in diagrams on paper. As a measure of structural knowledge, we subtracted the number of wrongly drawn relations from the number of correctly drawn relations and divided the difference by the number of all possible relations.

As a measure of strategy, we assessed VOTAT analogously to MicroDYN. A VOTAT event in Dynamis2 was defined by the manipulation of a single input variable, followed by at least five cycles (i.e., seconds) with no interventions. For a comprehensive measure of using the strategy, we calculated the proportion of input variables for which the VOTAT strategy was applied at least once in the exploration phases of each of the three scenarios. We averaged these proportions across the scenarios. Likewise, we defined a PULSE event by setting all input variables (back) to zero for at least five cycles and counted these events over all exploration runs. The reason why the operationalizations of PULSE differ between the two CDC tasks is that the scenarios in Dynamis2 are much longer than in MicroDYN. Due to the higher difficulty of Dynamis2 scenarios (longer runs, more dynamics), it can be quite reasonable to repeat PULSE interventions, for example to test hypotheses or to help memorizing certain effects.

### Design

We used a transfer design that allowed estimating transfer effects in both directions. As can be seen in Figure 2, there were four experimental conditions. In two conditions, subjects had two blocks of either MicroDYN (condition MM) or Dynamis2 (condition DD). Block 1 in these conditions consisted of separate Items that were not incorporated into the calculation of transfer effects. A third condition had one block of MicroDYN, followed by one block of Dynamis2 (condition MD). The fourth condition started with one block of Dynamis2, followed by one block of MicroDYN (condition DM). Participants were randomly assigned to one of the four conditions.

In MD, DM, and the second block in MM we applied six MicroDYN scenarios. In the first block of MM, the first scenario was declared as practice scenario. All blocks of Dynamis2 consisted of three scenarios (with a different set of scenarios in the first block of DD).

### Participants

One hundred-sixty-five subjects participated in the experiment. Students of diverse majors were recruited from the University of Heidelberg (*n* = 83) and from the University of Bayreuth (*n* = 82). Ethical approval was not required for this study in accordance with the national and institutional guidelines. Participation was in full freedom using informed consent.

We excluded three cases from the dataset due to dubious behavior during the experiment (not complying with the instructions; aborting the experiment). In other three cases, we imputed missing values of the variables Dynamis2 score or MicroDYN score. We applied multiple regression imputation based on the cases in the respective condition. The resulting dataset comprised *N* = 162 cases, 40 in the DD condition, 41 in DM, 42 in MD, and 39 in MM. The four conditions did not differ in figural reasoning, age and sex (all *Fs* < 1).

### Procedure

The experiment took place in two sessions. Session 1 began with a short introduction and the administration of the figural reasoning test with paper and pencil. Next, subjects worked on the three items of the Wason task. Session 1 ended with the first block of complex problem solving tasks, according to the design: either six items MicroDYN or three scenarios Dynamis2 (with two performance scores each). In Session 2, which took place 2 days after Session 1, we administered the second block of complex problem solving tasks, followed by two other tasks that are not reported in the present paper (a computerized in-basket task and an item from the wisdom questionnaire by Staudinger and Baltes, 1996). Each session lasted about 90 min.

## Results

For the statistical analyses, we used an alpha level of 0.05. In addition to the significance levels, we report Cohen's (1988) effect sizes or partial η^2^. The sample size was adequate for detecting at least medium-sized effects (*d* = 0.5) with a power of 0.72 for simple mean comparisons and a power of 0.68 for one-way ANOVA (Faul et al., 2007). Descriptive statistics of the most important variables are shown in Table 2.

To assess the reliability of the CPS measures, we calculated Cronbach's alpha values using the results of individual scenarios as items. We obtained α = 0.70 for the MicroDYN score (6 items), and α = 0.64 for the Dynamis2 score (6 items). The measure for figural reasoning, assessed with the extended WMT, yielded α = 0.75.

**Figure 4:**
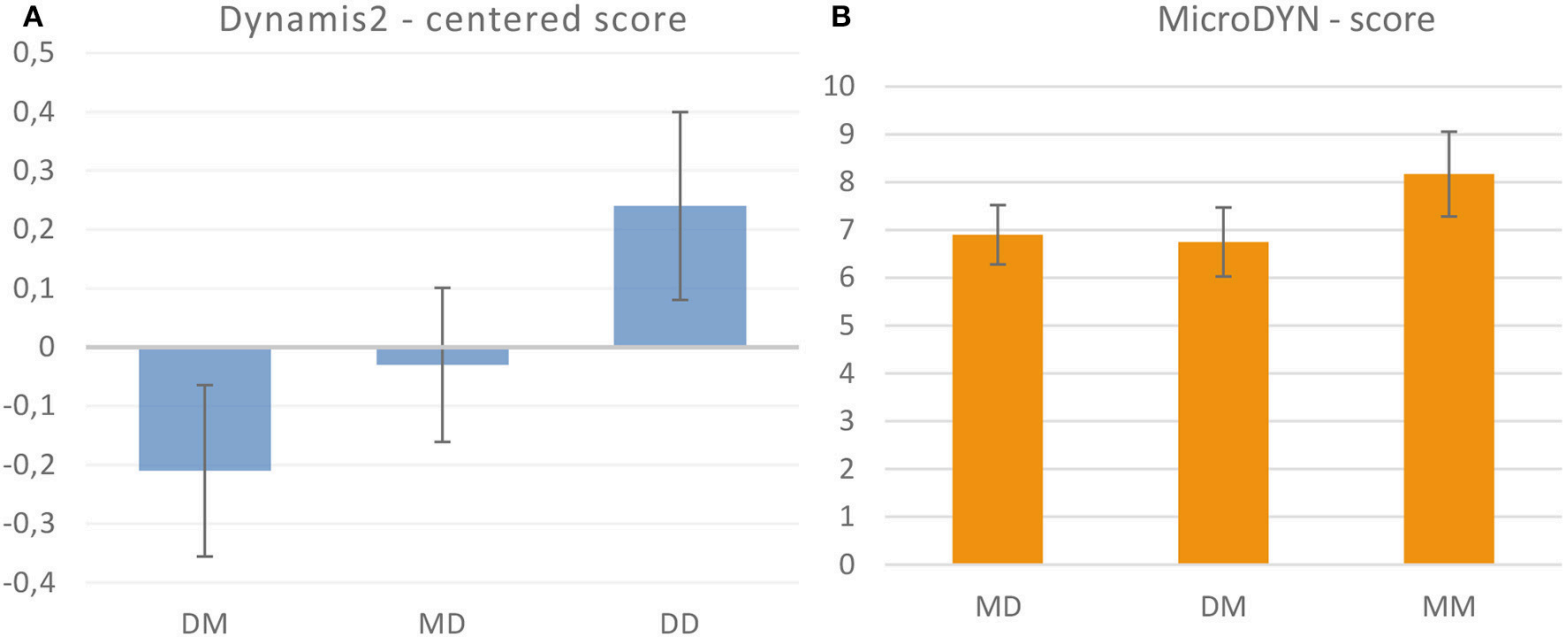
Means and 95% confidence intervals of the performance scores in Dynamis2 **(A)** and MicroDYN **(B)**. In the MM and DD conditions, the results of the second block are displayed.

Figure 4A shows the means of the Dynamis2 scores in the three conditions that involved Dynamis2 (error bars denote 95% confidence intervals). The value in the DD group denotes performance in Block 2. We found an overall effect of condition [*F*_(2, 121)_ = 9.11, *p* < 0.001, partial η^2^ = 0.132], with performance linearly increasing from the DM group to the DD group. A planned comparison between the DM and the MD group yielded a significant advantage of the MD group [*t*_(81)_ = 1.82, one-sided *p* < 0.05, *d* = 0.40]. This indicates that practicing MicroDYN in Block 1 is beneficial for Dynamis2. We calculated the amount of transfer using Katona's (1940) formula (Equation 2, cited after Singley and Anderson, 1989).

(2) $${\%}_{transfer}=\frac{C_{B1}-E_{B1}}{C_{B1}-C_{B2}}\times 100$$

The denominator of Equation (2) describes the amount of improvement when the same type of problem is solved a second time (*C* stands for control group, *E* for experimental group, *B* for the first and second occasion). The numerator describes the difference between the baseline performance (*C*_*B*__1_) and the performance of the experimental group in the target problem (where the experimental group has solved a different type of problem before). To estimate the transfer from MicroDYN to Dynamis, we used the mean performance in the first block of the DM group as baseline performance *C*_*B*__1_, performance in the second block of the DD group as *C*_*B*__2_, and performance in the second block of the MD group as *E*_*B*__1_. The calculation results in an estimate of 40% transfer from MicroDYN to Dynamis2. Hence, the part of Hypothesis 1 that assumed transfer is supported by the data.

A different picture emerges with the MicroDYN scores (Figure 4B). We found significant differences between the conditions [*F*_(2, 120)_ = 4.14, *p* < 0.05, partial η^2^ = 0.065], but no difference between the DM and the MD group [planned comparison, *t*_(81)_ = 0.32, two-sided *p* = 0.75, *d* = 0.07]. This means that as expected in Hypothesis 2, there is much less transfer from Dynamis2 to MicroDYN. Stating no transfer is not warranted because of the limited statistical power of our experiment.

### Prediction of dynamis2 performance

Table 1 shows the bivariate correlations between the performance measures, based on pairwise deletion (i.e., the largest possible part of the sample, respectively). For example, only three fourths of the sample have worked on MicroDYN (the MD, DM, and MM groups; other three fourths have worked on Dynamis2—the MD, DM, and DD groups). We found the expected significant correlations among figural reasoning and the two CPS tasks. Performance in MicroDYN and Dynamis2 are more closely related to each other than to figural reasoning. Performance in the Wason task, which is interactive like the CDC tasks, but not dynamic, correlates slightly, but mostly still significant with all other measures. The partial correlation between MicroDYN and Dynamis2 performance when figural reasoning is controlled for, is *r* = 0.422^**^.

**Table 1:** Bivariate correlation coefficients between various performance scores (^*^p < 0.05, ^**^p < 0.01).

	**Fig. reasoning**	**Wason**	**MicroDYN**
Wason	0.196^*^ (*n* = 160)		
MicroDYN	0.356^**^ (*n* = 122)	0.215^*^ (*n* = 121)	
Dynamis2	0.342^**^ (*n* = 123)	0.171 (*n* = 122)	0.465^**^ (*n* = 83)

**Table 2:** Descriptive statistics of important variables of the experiment in the four experimental conditions (R_t_: theoretical range; R_e_: empirical range; MDyn: MicroDYN; Dyn2: Dynamis2).

		**MD**			**DM**			**MM**			**DD**		
	**R_t_**	**M (s)**	***n***	**R_e_**	**M (s)**	***n***	**R_e_**	**M (s)**	***n***	**R_e_**	**M (s)**	***n***	**R_e_**
Performance MDyn	0.0–12.0	6.90 (2.06)	42	0.0–11.0	6.75 (2.36)	41	0.5–10.5	8.17 (2.81)	39	1.5–12.0			
Performance Dyn2	–	−0.03 (0.43)	42	−0.83–1.0	−0.21 (0.48)	41	−0.96–1.1				0.24 (0.52)	40	−1.47–1.26
VOTAT MDyn	0.0–1.0	0.85 (0.21)	42	0.12–1.0	0.91 (0.16)	41	0.35–1.0	0.85 (0.24)	39	0.12–1.0			
VOTAT Dyn2	0.0–1.0	0.89 (0.16)	42	0.44–1.0	0.82 (0.19)	41	0.33–1.0				0.94 (0.11)	40	0.56–1.0
PULSE MDyn	0.0–1.0	0.70 (0.35)	42	0.0–1.0	0.61 (0.35)	41	0.0–1.0	0.70 (0.38)	39	0.0–1.0			
PULSE Dyn2	0–20+	5.24 (3.88)	42	0–14	1.85 (2.47)	40	0–8				4.02 (3.91)	40	0–15
Figural Reasoning	0–20	14.7 (3.1)	42	6–20	14.2 (2.8)	41	8–19	14.1 (4.2)	39	3–20	14.7 (3.3)	40	6–20

To analyze how MicroDYN and figural reasoning predict performance in Dynamis2 we conducted a regression analysis and a commonality analysis (see Fischer et al., 2015a). These analyses are based on the part of the sample who worked on both MicroDYN and Dynamis2 (*n* = 83). Therefore, the bivariate correlation coefficients can differ from those shown in Table 1. The multiple regression coefficient is *R* = 0.54. Both predictors explain significant proportions of variance. The MicroDYN score explains a unique share of 15.4% variance (β = 0.402, *p* < 0.001); figural reasoning explains a unique share of 7.4% (β = 0.279, *p* < 0.01). The confounded variance explains 6.2% in the criterion. Altogether, these results support Hypothesis 3 that figural reasoning and MicroDYN predict performance in Dynamis2 (and that MicroDYN explains unique variance in Dynamis2, which suggests similar requirements).

### Mediation of transfer

To test our hypothesis that transfer from MicroDYN to Dynamis2 is mediated by use of the VOTAT strategy we checked three indicators. If all three indicators are positive, the hypothesis is confirmed.

Indicator 0 is a significant correlation between the amount of using the strategy and performance in Dynamis2. This is a basic requirement that is necessary but not sufficient for demonstrating a mediation. When there is no advantage of using a certain strategy, the strategy cannot be considered to explain a transfer effect.

Indicator 1 is a significant difference of the amount of using the strategy between the MD and the DM group. When the MD group has learned to use VOTAT in MicroDYN, then this group should use this strategy more often in Dynamis2 than the DM group who lacks this experience.

Indicator 2 provides a more challenging test of the hypothesis. It requires that there is a significant correlation between the use of the strategy in MicroDYN and performance in Dynamis2, particularly in the MD group.

As the correlation between use of VOTAT in Dynamis2 and performance in Dynamis2 is significant, but not substantial (*r* = 0.28^**^), Indicator 0 can be viewed as ambiguous and further tests will probably fail, because this indicator is essential. Indicator 1 is positive: There is a small, but significant difference in the use of the VOTAT strategy between the DM group (*M* = 0.82, *s* = 0.19) and the MD group [*M* = 0.89, *s* = 0.16, *t*_(81)_ = 1.88, one-sided *p* = 0.032, *d* = 0.46]. However, Indicator 2, the correlation between use of VOTAT in MicroDYN and performance in Dynamis2, *r* = 0.27 (MD group), does not support the hypothesis that transfer from MicroDYN to Dynamis2 is mediated through VOTAT. Hence, the part of Hypothesis 1 that refers to attributing the transfer to the use of VOTAT is not convincingly supported by the data.

To find an explanation of the transfer effect we searched for further strategic behaviors *post-hoc*. One of them is to set one or more input variables to values greater than zero, then setting all input variables back to zero for a specified number of time steps (one in MicroDYN, five in Dynamis2). This is a useful strategy for analyzing the momentum of the output variables. We dubbed this strategy “PULSE.” For quantifying this behavior, we counted how often PULSE occurred in all exploration rounds. For that variable, all indicators to mediation were positive: The correlation between PULSE and control performance in Dynamis2 is *r* = 0.40^**^ (Indicator 0); there are significant differences in the use of the strategy between the relevant groups [Indicator 1: DM group: *M* = 1.85, *s* = 2.47, MD group: *M* = 5.24, *s* = 3.88; *t*_(80)_ = 4.70, *p* < 0.001, *d* = 1.04]; and also the use of PULSE in MicroDYN correlates substantially with performance in Dynamis2 (Indicator 2: *r* = 0.46^**^ in the MD group). So the transfer from MicroDYN to Dynamis2 can partially be explained by the fact that many subjects have learned the strategy of deploying pulses in MicroDYN and applied it successfully to Dynamis2.

### Exploratory analyses

So far, the reported results largely support our hypotheses. As we also assessed structural knowledge in Dynamis2, using structural diagrams like those in MicroDYN, we could test further predictions of the preliminary process model^4^. If VOTAT or PULSE are important strategies for the acquisition of structural knowledge in Dynamis2, their use should correlate with the knowledge scores in each problem.

When we aggregated the scores across the three problems, the measures are correlated in the range of *r* = 0.35^**^ (PULSE—knowledge) to *r* = 0.41^**^ (knowledge—performance). When controlling for figural reasoning, the correlations are still significant (PULSE—knowledge: *r* = 0.35^**^, knowledge—performance: *r* = 0.39^**^).

When we look at the individual problems, the pattern becomes more ambiguous: The correlations between the number of PULSE events and the structural knowledge scores in three Dynamis2 problems are *r*_1_ = 0.11, *r*_2_ = 0.34^**^, and *r*_3_ = 0.12. The correlations between structural knowledge scores and performance in these problems are *r*_1_ = 0.25^**^, *r*_2_ = 0.48^**^, and *r*_3_ = 0.16. So the expected role of knowledge acquisition is corroborated only in Problem 2.

**Figure 5:**
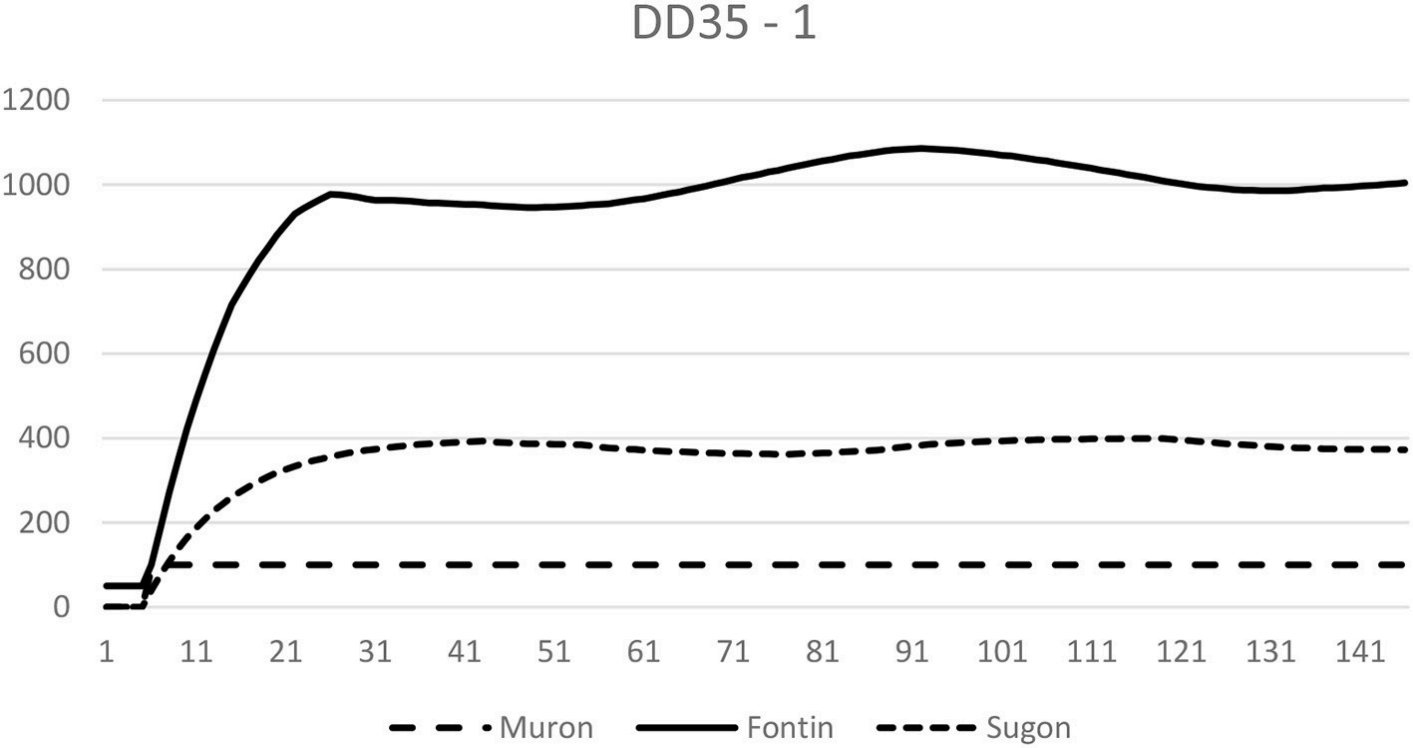
Course of output variables produced by a participant with low structural knowledge. Note that the participant still approached the goals well (Fontin = 1,000, Muron = 100). There were also participants with an inverted constellation: good structural knowledge and poor control performance.

This pattern of results may indicate that the low correlations in the single problems might have been due to reliability problems. However, overall this is not convincing evidence for an essential function of complete structural knowledge for performance in controlling dynamic systems. Correlations around *r* = 0.40 involve a noticeable number of cases that do not conform to the relation suggested by the coefficient. As an example, we depict in Figure 5 the progress of the system's variables of a participant with low structural knowledge (standard score *z* = −1.10) who nonetheless was successful in goal convergence (*z* = 1.68). The goals were Fontin = 1,000 and Muron = 100.

To compare our results with studies that were published after our experiment was run (e.g., Greiff et al., 2016), we report another *post-hoc* analysis of the correlations between strategy measures and performance in both CDC tasks. VOTAT and PULSE are more closely related in MicroDYN (*r* = 0.524^**^) than in Dynamis2 (*r* = 0.330^**^). The notion that using PULSE is more significant for successful problem solving in Dynamis2 than in MicroDYN is supported by the fact that the partial correlation between PULSE and performance in Dynamis2 controlling for VOTAT is only slightly lower (*r* = 0.344^**^) than the corresponding bivariate correlation (*r* = 0.401^**^). In MicroDYN, controlling for VOTAT changes the correlation from *r* = 0.615^**^ to *r* = 0.410^**^.

## Discussion

By and large, our hypotheses are supported by the data: Performance in MicroDYN explains a unique proportion of variance in Dynamis2. We found positive transfer from MicroDYN to Dynamis2, but not in the opposite direction. This null result has to be interpreted with the reservation that the statistical power of the respective test was rather low (0.72). It may be that studies with larger samples could detect transfer effects from Dynamis2 to MicroDYN. However, the asymmetry of the transfer effects is obvious in our experiment. The assumption that transfer was mediated by using VOTAT was not clearly supported; instead, it was a different strategy called PULSE that could explain the transfer effect. PULSE is defined by setting input variables to zero and observing the system for a number of time steps (≥1 in MicroDYN and ≥5 in Dynamis2). This strategy—Greiff et al. (2016) refer to it as “non-interfering observation behavior”—is helpful for identifying eigendynamics (Schoppek and Fischer, 2015).

Exploratory analyses have shown that the relationships between using PULSE and the resulting structural knowledge, as well as between the latter and control performance are not as close as one might expect. Only when the respective scores were aggregated, we found substantial correlations.

With regard to aggregated results, our findings can be interpreted as supporting the standard model of CPS (Fischer et al., 2012), which assigns a critical role to knowledge acquisition (and strategies for acquiring knowledge) for the control of complex dynamic systems. As this has been shown before repeatedly (Funke, 1992; Osman, 2008; Greiff et al., 2012; Wüstenberg et al., 2012), we also want to discuss the controversial details and limitations of our findings later on. Another positive statement is that MicroDYN was successfully validated. Explaining a unique proportion of 15.4% variance in Dynamis2 performance is a considerable accomplishment, given the differences between these two classes of problems: More dynamics and momentum in Dynamis2, real-time vs. user-controlled course of events, 250 vs. on average 8 time steps (median). Also, consider the fact that the measures in the present study are manifest variables, whereas many comparable studies report proportions based on latent variables, which raises the amount of explained variance. For example, with regard to latent variables Greiff et al. (2015) report a variance overlap of 24% between MicroDYN and MicroFIN after partialling out figural reasoning (MicroFIN is another class of minimal complex systems, based on finite automata, but administered in a way similar to MicroDYN, cf. Greiff et al., 2013). Between MicroDYN and Tailorshop, they report an overlap of 7%. However, the respective study has been criticized for several methodological shortcomings, such as having administered the Tailorshop inadequately, namely in one round without a separate exploration phase (Funke et al., 2017; Kretzschmar, 2017). Altogether, the variance overlap between MicroDYN and Dynamis2 (on top of the variance that both tasks share with figural reasoning) fits neatly within the range of values from comparable studies.

In recent studies, it turned out that the established finding that MicroDYN explains variance in school grades over and above figural reasoning, cannot be replicated when intelligence is operationalized broadly (Kretzschmar et al., 2016; Lotz et al., 2016). This casts doubt on the distinctiveness perspective that construes CPS as an ability separate from general intelligence (Kretzschmar et al., 2016). However, Kretzschmar et al. (2016) still found unique covariance between MicroDYN and MicroFIN not attributable to intelligence, which can be viewed as supporting the distinctiveness view. Consistent with this, we also found considerable unique covariance between the two different CDC tasks. Irrespective of the difficult question if CPS should be construed as an ability construct in its own right, our results clearly confirm the notion that figural reasoning facilitates complex problem solving.

From a practical perspective, our results suggest that MicroDYN can be used as training device for more dynamic task environments. However, as there are numerous instances of rather ineffective CPS training (e.g., Schoppek, 2002, 2004; Kretzschmar and Süß, 2015) this prediction needs to be confirmed in further studies. We shall discuss the question what kind of real life situations are modeled by MicroDYN or Dynamis2 below.

The finding that not VOTAT could explain the transfer effect from MicroDYN to Dynamis2 but the related PULSE tactic points to the plurality of potentially relevant tactics or strategies. *Post-hoc* analyses showed that our findings correspond with recent analyses by Greiff et al. (2016), who found that controlling for VOTAT substantially reduces the relation between PULSE and knowledge acquisition in MicroDYN. However, we did not find this pattern of results in Dynamis2, where PULSE plays a discrete role. We consider two possible explanations for this difference: First, whereas all Dynamis2 scenarios involved eigendynamics, this was the case for only half of the MicroDYN scenarios (which is common practice in research with MicroDYN). Second, the real-time character of Dynamis2 makes it more obvious to vary only one variable at a time (even though it was possible to vary more variables, because the input values were transferred to the running simulation only when an apply button was pressed). Maybe a certain proportion of VOTAT events in Dynamis2 was not actually analyzed by the participants, but rather happened as a byproduct of their way of handling the CDC environment.

Findings like these raise questions about the generality of problem solving strategies: If the viability of strategies such as VOTAT and PULSE differs between certain problem classes, they could be used for classifying complex problems. Many studies have confirmed the significance of VOTAT for scientific reasoning as well as for CDC tasks from the Dynamis family (Vollmeyer et al., 1996; Chen and Klahr, 1999; Wüstenberg et al., 2014). Our results are an exception to this series, as they highlight the importance of PULSE. However, on a conceptual level the PULSE strategy is closely related to VOTAT and could be considered an extension to that strategy. On the other hand, there are many CDC tasks in- and outside the laboratory that obviously cannot be accomplished using experimental tactics like VOTAT. For example, when pilots have to handle an in-flight emergency, they are not well advised to adopt a VOTAT strategy. Generally, VOTAT is not an option in situations that forbid free exploration. In the discussion about the relationship between strategies and complex problems we should keep in mind that there are good arguments that most problem solving strategies are domain-specific to some extent (for a discussion see Tricot and Sweller, 2014; Fischer and Neubert, 2015).

Although correlations around *r* = 0.41 (e.g., between knowledge and performance) are usually interpreted as supporting an assumed causal relation, they leave a large amount of unexplained variance, and the number of cases that differ from the general rule is not negligible. In our context, this means that there are subjects who do control our systems successfully with merely rudimentary structural knowledge. To date, most authors have taken a stand on the question about the significance of structural knowledge for performance in system control—either approving (Funke, 1992; Osman, 2008; Greiff et al., 2012; Wüstenberg et al., 2012) or disapproving (Broadbent et al., 1986; Berry and Broadbent, 1988; Dienes and Fahey, 1998; Fum and Stocco, 2003). In our opinion, the evidence on this question is so ambiguous that an all-or-none answer is not appropriate. Some subjects seem to rely on structural knowledge, some don't (see Figure 5). Therefore, future research and theorizing should be aimed at specifying situational and individual conditions that predict the use (or usefulness) of structural knowledge^5^. As mentioned in the introduction, we believe that available working memory capacity—either varied individually or situationally (concurrent tasks, fatigue) could be such a predictor: The lower the capacity, the less promising a WM-intensive strategy is. For an excellent example of this idea applied to a static problem, see Jongman and Taatgen (1999). Although our results are consistent with these WM-related assumptions, they are not adequate for testing them directly. We plan to do this in future experiments.

If structural knowledge is not the exclusive necessary condition for successful system control, what other forms of knowledge are relevant? At this point, we can only speculate, based on our experience in the domain: Knowledge about and experience with growth and decay processes, saturation, and time delays are in our view concepts that are worth investigating. Relatedly, concepts such as wisdom may foster an appropriate way of controlling complex and dynamic systems (Fischer, 2015; Fischer and Funke, 2016).

In real life, situations where problem solvers have to find out the causal structure of a system through systematic exploration are rare. Comparable settings can be found in scientific discovery, pharmaceutical efficacy studies, organizational troubleshooting (Reed, 1997), or psychotherapy. On the other hand, there are quite a lot of situations where dynamically changing variables have to be controlled: driving a car, heating a house economically, controlling combustion processes, or monitoring vital functions in intensive care. Therefore, we consider it worthwhile to investigate how humans handle dynamic systems. However, to make our research more applicable, we—the scientific community—should shift the focus away from questions about the acquisition of structural knowledge about simple artificial systems to questions about how humans approach more realistic CDC tasks with existing knowledge that may be limited or simplified. For example, Beckmann and Goode (2014) found that participants overly relied on their previous knowledge when dealing with a system that was embedded in a familiar context.

At last, we should not forget that although Dynamis2 exceeds MicroDYN in complexity and dynamics, both environments share some family resemblance. Therefore, we cannot generalize our results to CPS in general. Future research is necessary to investigate the common requirements of systems of the Dynamis type and more semantically rich systems such as the Tailorshop, where knowledge acquisition does not play the same role as in MicroDYN (Funke, 2014). We believe that transfer experiments could play an important role in answering these questions, too.

## Ethics statement

This study was carried out in accordance with the recommendations of “Ethische Richtlinien der Deutschen Gesellschaft fur Psychologie e.V. und des Berufsverbands Deutscher Psychologinnen und Psychologen e.V”. In accordance with the guidelines of the ethical committee at the University of Bayreuth, the study was exempt from ethical approval procedures because participation was in full freedom using informed consent and the materials and procedures were not invasive.

## Author contributions

WS planned and conducted the reported experiment together with AF. The report was written mainly by the first author, with some support from the second author, who contributed a few sections. Both authors discussed and revised the text together.

### Conflict of interest statement

The authors declare that the research was conducted in the absence of any commercial or financial relationships that could be construed as a potential conflict of interest.

## Footnotes

^1^Although we would prefer to distinguish between tactics (= concrete methods for accomplishing goals), and strategies (= abstract plans), we use the more general term “strategy” for both, because the distinction has not become widely accepted in cognitive science.

^2^A side effect is an effect of one output variable on another; eigendynamic is the effect of an output variable on itself (Funke, 1992). Considerations about how to deal with eigendynamic can be traced back to the early days of CPS (Dörner, 1980; Beckmann, 1994; Dörner and Schaub, 1994).

^3^Problems that are used for learning in a transfer design are called source problems; problems in the transfer phase are called target problems. Combining refers to the fact that all levels of the factors “CPS environment” and “Function (source vs. target)” were combined.

^4^We report this “under exploratory analyses”, because we had not put forward this hypothesis ex ante. In view of the current debate about false-positive results in psychological research (Pashler and Wagenmakers, 2012; Ulrich et al., 2016), we attach much importance to clearly distinguishing between the context of discovery and the context of justification.

^5^This endeavor could well be tackled in the spirit of the early studies of Broadbent and colleagues. However, we suspect that the “salience” concept these authors focused on is closely tied to the very special characteristic of oscillatory eigendynamics, rather than a generalizable determinant of structural knowledge (see also Hundertmark et al., 2015).
