# Supplementary material for: Common Process Demands of Two Complex Dynamic Control Tasks: Transfer Is Mediated by Comprehensive Strategies
Source: Front Psychol. 2017 Dec 19;8:2145. doi: 10.3389/fpsyg.2017.02145 (PMC5742126; doi:10.3389/fpsyg.2017.02145)

## Appendix

For the sake of readability, we omitted indices in the following equations. Output variables (Y) on the left side of the equation denote current values (*t*); output variables on the right side denote values from the previous cycle (*t*-1). Input variables (X) always denote current values.

### MicroDYN Items

Items 5-10 constituted the MicroDYN block in the conditions MD and DM, and the second block in condition MM. Items 1-5 and 10 constituted the first block in MM.

| Item 1  Y1 = Y1  Y2 = Y2 + X1 | Item 6  Y1 = Y1 + 2∙X1  Y2 = Y2 + 2∙X2 + 2∙X3  Y3 = Y3 + 2∙X3 |
| --- | --- |
| Item 2  Y1 = Y1 + 2∙X2  Y2 = Y2 + 2∙X2 | Item 7  Y1 = Y1  Y2 = 1.33∙Y2 + 2∙X1 + 2∙X2  Y3 = Y3 + 2∙X3 |
| Item 3  Y1 = 1.05∙Y1 + 2∙X1  Y2 = Y2 + 2∙X2  Y3 = Y3 + 2∙X2 | Item 8  Y1 = 1.33∙Y1 + 2∙X1  Y2 = Y2 + 2∙X3 |
| Item 4  Y1 = Y1 + 2∙X1 + 2∙X2  Y2 = Y2 + 2∙X3 | Item 9  Y1 = Y1 + 2∙X2  Y2 = Y2 + 2∙X1  Y3 = Y3 + 2∙X3 |
| Item 5 (used with 2 cover stories)  Y1 = Y1 + 2∙X1 + 2∙X2  Y2 = Y2 + 2∙X2  Y3 = Y3 + 2∙X3 | Item 10 (used with 2 cover stories)  Y1 = Y1 + 2∙X1  Y2 = Y2 + 2∙X1  Y3 = 1.33∙Y3 + 2∙X2 |

Figure 6: Screenshot of a MicroDYN scenario.


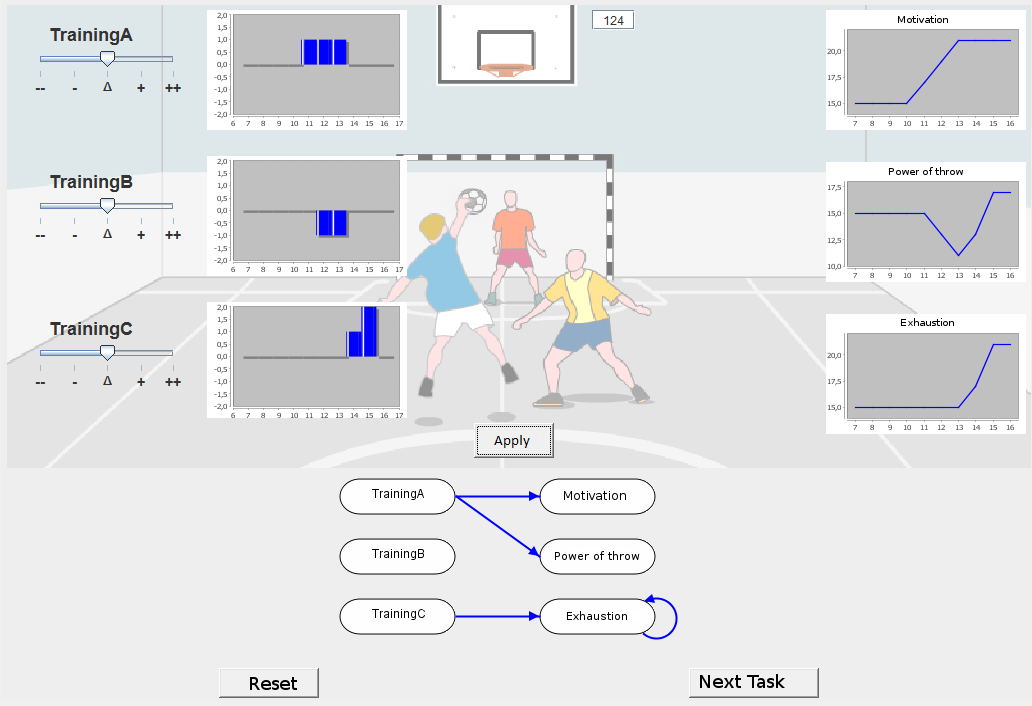


### Dynamis2 Items

The first three items constituted the Dynamis2 block in the conditions MD and DM, and the second block in condition DD. The remaining items constituted Block 1 in DD.

#### Disco

Rocker = 0.5∙Rocker + Rock - Classic

Raver = 0.5∙Raver + Rave - Classic

Minors = 0.5∙Minors + 0.2∙Rock + 0.2∙Rave -10∙Classic

#### Soup (Output variables mean satisfaction of the respective person with meals)

Peter = Peter + Saus - Veg

Monika = Monika + Veg - Nood

Fritz = Fritz + 2∙Saus - 2∙Veg

#### Medicines

Muron = 0.1∙Muron + 2∙MedA

Fontin = Fontin + 0.5∙Muron - 0.2∙Sugon + MedB

Sugon = 0.9∙Sugon + MedC

#### Gecko

Activity = 0.5∙Activity + 1.25∙Temp - 0.75∙Noise

Color = 0.2∙Color + 1.75∙Humid

Costs = 0.8∙Temp + 0.4∙Humid - Noise

#### Flowers (Output variables mean satisfaction of the respective person with floral decoration)

Luise = Luise + 5∙Daffodills

Tanja = Tanja - Tulips + Roses

Lena = Lena + Tulips – Roses

#### Vegetables

Beancrop = Beanseed + 0.8∙Beancrop - 0.2∙Peacrop - 0.1∙Fennelcrop

Peacrop = Peaseed - 0.2∙Beancrop + 0.8∙Peacrop - 0.15∙Fennelcrop

Fennelcrop = Fennelseed - 0.05∙Beancrop - 0.05∙Peacrop + 0.8∙Fennelcrop

Figure 7: Screenshot of a Dynamis2 scenario.


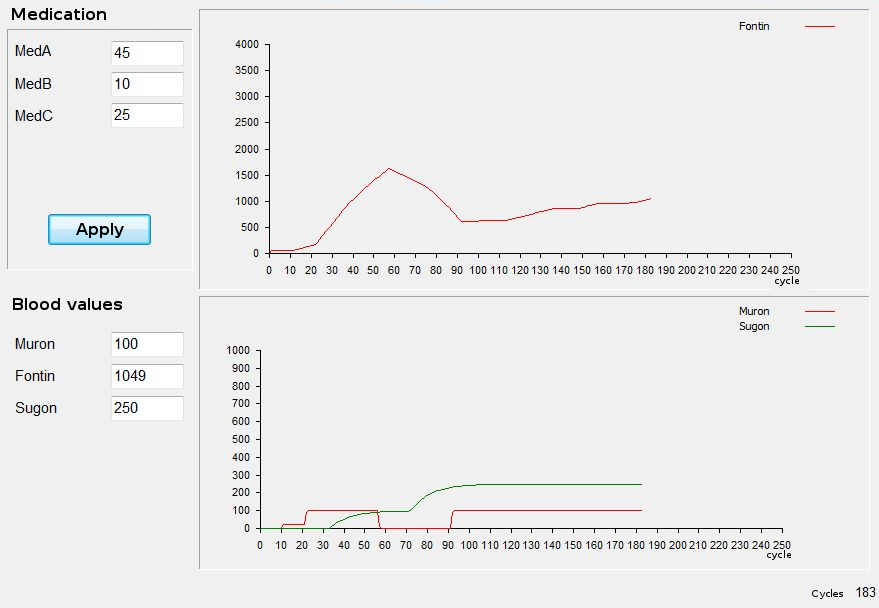

Supplement: Supplementary file 1 [file Appendix.docx]
